# Bioinformatics-driven single-cell sequencing analysis of angiogenesis-related genes in rheumatoid arthritis

**DOI:** 10.3389/fimmu.2026.1789899

**Published:** 2026-07-15

**Authors:** Xiaolan Shen, Guibin Deng, Xiang Guo, Xiaoman Liu, Xiaoqiang Hou, Zhitao Feng

**Affiliations:** 1 Third-grade Pharmacological Laboratory on Traditional Chinese Medicine,State Administration of Traditional Chinese Medicine, China Three Gorges University, Yichang, China; 2 College of Medicine and Health Sciences, China Three Gorges University, Yichang, China; 3 The First College of Clinical Medical Sciences, China Three Gorges University, Yichang, Hubei, China; 4 Yichang Huimin Hospital, Yichang, China; 5 The Second Clinical Medical School, Guangzhou University of Chinese Medicine, Guangzhou, China

**Keywords:** angiogenesis, bioinformatics, disease activity, rheumatoid arthritis, single-cell sequencing analysis

## Abstract

**Objective:**

To explore angiogenesis-related genes and immune cell profiles in RA and assess their clinical relevance in RA patients.

**Methods:**

Gene expression datasets(GEO) of RA synovium were retrieved from the public database, and differential gene and immune infiltration analysis were performed using R. Angiogenesis-related genes were obtained from the Gene Set Enrichment Analysis(GSEA) database, and Receiver Operating Characteristic(ROC) curves were used to identify diagnostic genes based on their expression in RA synovium. Gene Ontology (GO) functional and Kyoto Encyclopedia of Genes and Genomes (KEGG) pathway analyses were conducted using the DAVID and Metaspace database. Single-cell sequencing data related to RA were analyzed for dimensionality reduction and cell type annotation, with core genes identified. Finally, PBMCs from RA clinical samples were collected for Quantitative Real-Time PCR (RT-qPCR) and clinical correlation analysis.

**Results:**

This study identified 11 core genes associated with synovium and angiogenesis in RA. Notably, the diagnostic accuracy rates of IL10, E2F7, and E2F8 were found to be relatively high. Furthermore, we observed that these genes were significantly enriched in key signaling pathways such as HIF1 and VEGFA. Consequently, we determined that HIF1, VEGFA, E2F7, E2F8, and IL-10 are critical factors contributing to RA-related angiogenesis, with a significant correlation noted for the HIF1 signaling pathway. Additionally, our research indicates that various immune cell types, including B cells, plasma cells, T cells, natural killer (NK) cells, monocytes, macrophages, quiescent dendritic cells, mast cells, and neutrophils-are all implicated in the pathogenesis of RA. Relevant analyses demonstrate a positive correlation between HIF1 and IL10 as well as E2F7 and E2F8. However, the relationship between HIF1/VEGFA and common differential immune cell populations such as memory B cells, plasma cells, and activated memory CD4^+^ T cells is generally low. Further exploration of the clinical significance of the core target revealed that the mRNA levels of E2F7, E2F8, IL10, HIF1, and VEGFA in RA patients were significantly higher than those in the healthy control group. Among them, the levels of HIF1, VEGFA and E2F8 in patients with active RA were significantly higher than those in other periods, and their expression levels were positively correlated with the disease activity. In contrast, although the levels of E2F7 and IL10 in RA patients in remission decreased significantly, they were still higher than those in the normal control group.

**Conclusion:**

The study pinpointed HIF1, VEGFA, E2F7, E2F8, and IL-10 as pivotal angiogenic genes whose expression tightly mirrors clinical disease activity. Notably, HIF1, VEGFA, and E2F8 escalate in parallel with increasing severity, are shaped by the inflammatory synovial milieu, and appear to fuel both angiogenesis and RA progression.

## 1 Background

Rheumatoid Arthritis (RA) is a chronic, systemic autoimmune disease that primarily manifests as persistent joint inflammation, leading to joint swelling, pain, and functional impairment (1–3). According to studies, RA is widespread globally and affects about 1% of the adult population, with female prevalence being two to three times that of males (4, 5). The pathogenesis of RA is complex and not yet fully understood. However, existing studies indicate that angiogenesis plays a critical role in RA development (6, 7). Angiogenesis, the process of forming new capillaries from existing blood vessels, is significant in RA synovitis and bone destruction (8, 9). The process is initiated when vascular endothelial growth factor (VEGF) and fibroblast growth factor (FGF) bind to receptors on endothelial cells, triggering their activation. Activated endothelial cells induce the breakdown of tight junctions, increasing vascular permeability. Matrix metalloproteinases (MMPs) subsequently degrade the vascular basement membrane, promoting endothelial cell migration, proliferation, and new blood vessel formation (10, 11). These newly formed vascular networks are regulated by pro-angiogenic factors, enhancing the recruitment of surrounding cells and promoting blood flow, thereby supporting the oxygen and nutrient supply to synovial cells. This process further drives the progression of synovitis and the invasive destruction of joints (12, 13). Additionally, hypoxia-inducible factor 1 (HIF1) plays a key role in this pathological process, with VEGFA serving as a critical downstream effector molecule of HIF1. This promotes further activation, proliferation, and migration of endothelial cells, supporting the high metabolic demands of inflamed joints and potentially facilitating the infiltration of inflammatory cells and the transmission of inflammatory mediators (14, 15). However, the clinical relevance of angiogenesis-related genes (ARGs) in RA has not been fully elucidated. Therefore, further identification of the biological mechanisms underlying angiogenesis in RA and the discovery of new biomarkers for targeted anti-angiogenic therapy are crucial to improving RA management.

**Figure 1:**
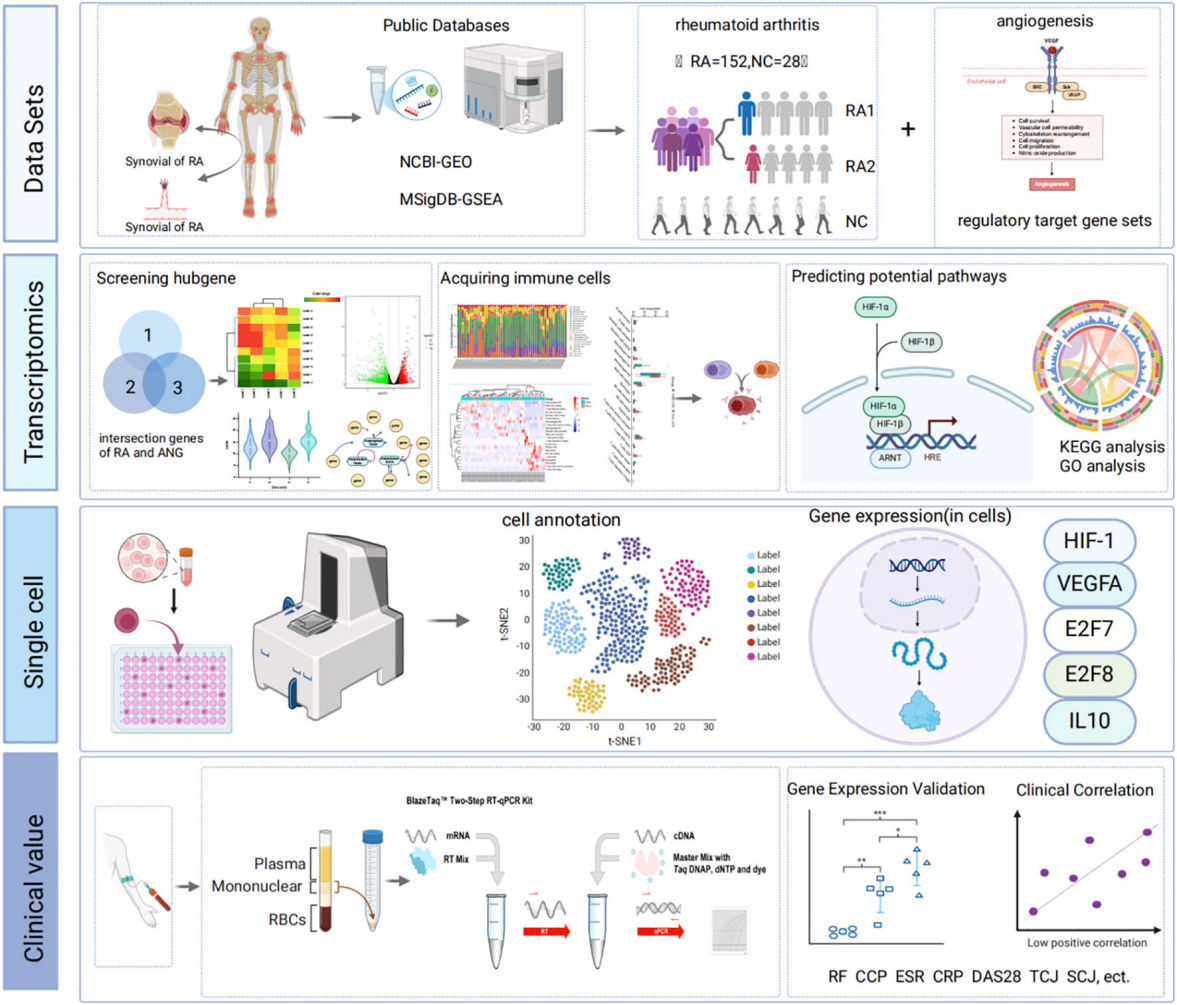
Workflow of the study. **(A)** Retrieval of RA and angiogenesis-related datasets from public databases. **(B)** Comprehensive analysis of angiogenesis-related genes in RA, including associated signaling pathways and immune cell infiltration characteristics. **(C)** Single-cell transcriptomic analysis to determine the expression profiles of core target genes within specific cell populations. **(D)** Collection of peripheral blood mononuclear cells (PBMCs) from clinical RA patients for quantitative mRNA expression analysis of target genes, alongside acquisition of RA-associated clinical indicators to assess clinical relevance.

By utilizing bioinformatics tools, researchers can analyze large genetic datasets to identify key genes and regulatory networks associated with RA (16). This interdisciplinary approach not only deepens our understanding of RA pathogenesis but also offers potential for the development of new therapeutic targets, particularly in controlling the critical process of angiogenesis. Therefore, research focusing on RA-related genes through bioinformatics and Single-Cell Sequencing Analysis provides new perspectives and tools for precision medicine in RA.The workflow of the study is shown in **Figure 1**.

## 2 Methodology

### 2.1 Data collection and processing

RA joint synovial gene expression microarray datasets were retrieved from the publicly available Gene Expression Omnibus (GEO) database (https://www.ncbi.nlm.nih.gov/geo/) using the keywords “rheumatoid arthritis” and “joint synovial”. Inclusion criteria for datasets were as follows: (i) species specified as human; (ii) tissue origin from joint synovium; (iii) datasets must include both normal control samples and RA samples, with a minimum of three samples per group; (iv) all samples must be free from other diseases, and RA samples must not have undergone drug or other interventions; (v) datasets must have complete information, including sequencing chip details and relevant clinical data. Differential expression analysis was conducted using RStudio software. Probe names were converted to gene names according to chip annotation data, with average values taken for genes associated with multiple probes, and redundant probes removed. Gene expression data normalization was performed using the “limma” R package.

Additionally, the “c5.all.v7.1.symbols.gmt” gene functional dataset was downloaded from the MSigDB database (https://www.gsea-msigdb.org/gsea/index.jsp), and genes related to “angiogenesis” were identified to construct an angiogenesis gene set for subsequent analysis.

### 2.2 Consensus clustering analysis of RA synovial tissue samples

Based on gene expression profiles from RA synovial tissue datasets, consensus clustering analysis was performed using the ConsensusClusterPlus package in R. The cumulative distribution function (CDF) was utilized to evaluate the area under the curve, aiming to maximize it. Simultaneously, the number of clusters was determined by assessing the rate of decline in the CDF delta area, with a preference for the slowest decline. Balancing these two criteria, the optimal number of clusters was selected. The clustering result with the highest average consensus within clusters was considered optimal for classifying RA synovial tissue samples into distinct molecular subtypes based on gene expression patterns. Based on the RA synovial gene expression matrix retrieved from the GEO database, the expression levels of the angiogenesis-related shared DEGs were measured across RA subgroups (RA1, RA2) and the normal control (NC) group. Hierarchical clustering was performed using RStudio to group samples based on gene expression patterns, allowing assessment of similarities in expression profiles among the different groups.

### 2.3 Differential gene expression analysis of RA synovial tissue

Differential gene expression analysis of RA synovial tissues and their molecular subtypes was conducted using R software to identify differentially expressed genes (DEGs) between various comparison groups and controls. Based on the platform’s annotation file, probe IDs were converted to gene symbols. For genes with multiple probes, the average expression value was taken, and redundant probes were removed. The gene expression data were log2-transformed and normalized using the limma package in R. Group-wise comparisons were performed as per the analytical design. DEGs were identified using a threshold of *P* < 0.05 and absolute fold change (FC) > 2. The significance of gene expression differences was visualized using volcano plots.

### 2.4 Intersection analysis of shared genes

Venn diagram analysis was performed using the online tool (http://www.interactivenn.net/) to identify the overlapping genes between DEGs obtained from differential expression analysis, RA molecular subgroups, and angiogenesis-related genes. The overlapping genes were visualized using Venn diagrams, and the common DEGs were considered angiogenesis-related genes potentially involved in RA pathogenesis. The expression levels of the identified angiogenesis-related shared DEGs were validated in the GSE89408 dataset by comparing RA and NC groups. The differences in gene expression were visualized using violin plots, and statistical significance was annotated (*P* < 0.05), demonstrating gene-specific differential expression between RA patients and healthy controls.

### 2.5 Diagnostic value analysis

The diagnostic value of the angiogenesis-related shared DEGs was evaluated using the R package pROC (version 1.17.0.1). Receiver Operating Characteristic (ROC) curve analysis was performed to calculate the area under the curve (AUC), assessing the ability of the shared DEGs to distinguish RA patients from normal controls and reflecting their potential as diagnostic biomarkers.

### 2.6 Functional and pathway enrichment analysis

The official gene symbols of the angiogenesis-related shared DEGs were submitted to the Database for Annotation, Visualization, and Integrated Discovery (DAVID; https://david.ncifcrf.gov/) and Metascape (https://metascape.org/) for Kyoto Encyclopedia of Genes and Genomes (KEGG) pathway and Gene Ontology (GO) enrichment analysis. The species was set as Homo sapiens and the identifier type as “OFFICIAL_GENE_SYMBOL.” Enrichment results were filtered using a significance threshold of *P* < 0.05. The enriched biological processes and signaling pathways potentially involved in RA angiogenesis were visualized using bubble plots.

### 2.7 Immune infiltration analysis

Using R software and the principle of support vector regression, the gene expression matrix of synovial samples was deconvoluted into the relative abundance of 22 immune cell types. The CIBERSORT algorithm (https://cibersort.stanford.edu/) was applied to estimate the infiltration levels of naïve B cells, memory B cells, plasma cells, CD8^+^ T cells, memory CD4^+^ T cells, regulatory T cells (Tregs), NK cells, monocytes, M0/M1/M2 macrophages, resting/activated dendritic cells, mast cells, eosinophils, and others. The abundance of each immune cell type was compared across different sample groups to explore immune landscape alterations in RA.

### 2.8 Correlation analysis

Correlation analysis was performed using the corrplot package in R to evaluate the associations between core genes from enriched pathways, angiogenesis-related DEGs, and infiltrating immune cell types. Pearson correlation coefficients were calculated and visualized in a correlation matrix, with a color gradient used to indicate the strength and direction of correlations.

### 2.9 Single-cell transcriptomic analysis

Single-cell RNA sequencing data from RA patients (GSE159117) were retrieved from the GEO database. Data preprocessing and quality control were performed in R. Repeated genes were averaged, and the dataset was converted into a Seurat object. The percentage of mitochondrial genes was calculated using the “PercentageFeatureSet” function, and cells with <20% mitochondrial gene content and >500 expressed genes were retained. The filtered dataset was then used for downstream analyses. Dimensionality-reduced data were clustered using the t-distributed stochastic neighbor embedding (t-SNE) method. Cell types were annotated using cellmarker 2.0, and the expression of core genes was quantified within each cluster. Based on the expression patterns of core genes, *k*-means clustering was used to further classify cells into distinct groups.

### 2.10 Clinical validation

#### 2.10.1 Clinical sample collection

Approved by the Medical Ethics Committee of Yichang Central People’s Hospital (Approval No. 2023-246-01), and all participants signed written informed consent. Peripheral blood samples were collected from 6 normal control (NC) and 12 patients clinically diagnosed with RA at Yichang Central People’s Hospital. Peripheral blood mononuclear cells(PBMCs) were isolated using a dedicated extraction kit and stored at -80°C for subsequent analyze.

Inclusion criteria:(i) RA patients diagnosed according to the 2010 ACR/EULAR classification criteria for RA;(ii) Patients in the active phase of RA;(iii) Age between 18 and 80 years;(iv) No gender restriction;(v) Signed informed consent.

Exclusion criteria:(i) Individuals with psychiatric disorders or unable to cooperate for other reasons;(ii) Presence of other acute or chronic comorbidities;(iii) Women who are pregnant, breastfeeding, or planning to conceive in the near term;(iv) Patients who had received biological agent therapy within 4 weeks prior to enrollment.

#### 2.10.2 Quantitative real-time PCR

PBMCs were isolated from peripheral blood samples of RA patients and healthy controls using a density gradient centrifugation method. Total RNA was extracted from the PBMCs using the TRIzol reagent, following standard protocols. Genomic DNA was removed during the extraction process. After quantifying RNA concentration and purity, 1000 ng of RNA was used as the template for reverse transcription to synthesize 20μL of complementary DNA (cDNA).

Primers were diluted with DNase/RNase-free deionized water and stored at -20°C as working solutions. Real-time quantitative PCR (qRT-PCR) was performed using SYBR Green chemistry in a 20 μL reaction volume, with 40 amplification cycles. Each reaction tube contained 5-fold diluted cDNA, 10-fold diluted forward and reverse primers, ROX reference dye, DNase/RNase-free water, and double-distilled water, all prepared according to the kit instructions. After brief centrifugation using a mini centrifuge, reactions were run on a real-time PCR system.

Amplification curves, melting curves, and cycle threshol d (Ct) values were obtained for each sample. Ct values greater than 35 were excluded from the analysis. glyceraldehyde-3-phosphate dehydrogenase (GAPDH) was used as the internal control. Each sample was analyzed in duplicate wells, and the experiment was repeated three times. Primer sequences are listed in **Table 1**. Relative gene expression levels were calculated using the 2^▲▲Ct^ method. The primer sequences are shown in **Table 1**.

**Table 1:** Primer sequences of genes.

Gene	Primer sequences(5'-3')
GAPDH	Forward:GGAAGCTTGTCATCAATGGAAATC
	Reverse : TGATGACCCTTTTGGCTCCC
IL10	Forward : GCTGAGAACCAAGACCCAGACAT
	Reverse : GCATTCTTCACCTGCTCCACG
E2F7	Forward : GCTCGCTATCCAAGTTATCCCT
	Reverse : CATACTGATTCTTAGCCACCCG
E2F8	Forward : GTGAATAATGACATCTGCCTTGACG
	Reverse : CTCTTCAAGGTGCCAAGGGTT
HIF1	Forward : TGATTGCATCTCCATCTCCTACC
	Reverse : GACTCAAAGCGACAGATAACACG
VEGFA	Forward : CCCACTGAGGAGTCCAACATC
	Reverse : TACACGCTCCAGGACTTATACCG

#### 2.10.3 Clinical correlation analysis

Clinical information collected from RA patients included gender, age, tender joint count (TJC), swollen joint count (SJC), erythrocyte sedimentation rate (ESR), C-reactive protein (CRP), Disease Activity Score in 28 joints (DAS28), rheumatoid factor (RF), and anti-cyclic citrullinated peptide antibodies (anti-CCP).

The mRNA expression levels of HIF1, VEGFA, E2F7, E2F8, and IL-10, as determined by RT-qPCR, were analyzed for correlations with these clinical parameters.

### 2.11 Statistical analysis

Study data were analyzed using SPSS27.0.1 software. Results are expressed as mean ± standard deviation ( $\bar{\text{X}}\;\pm \;\text{S}$). Comparisons between two groups were performed using the *t*-test, with *P* < 0.05 considered statistically significant.

## 3 Results

### 3.1 Differential expression analysis

**Figure 2:**
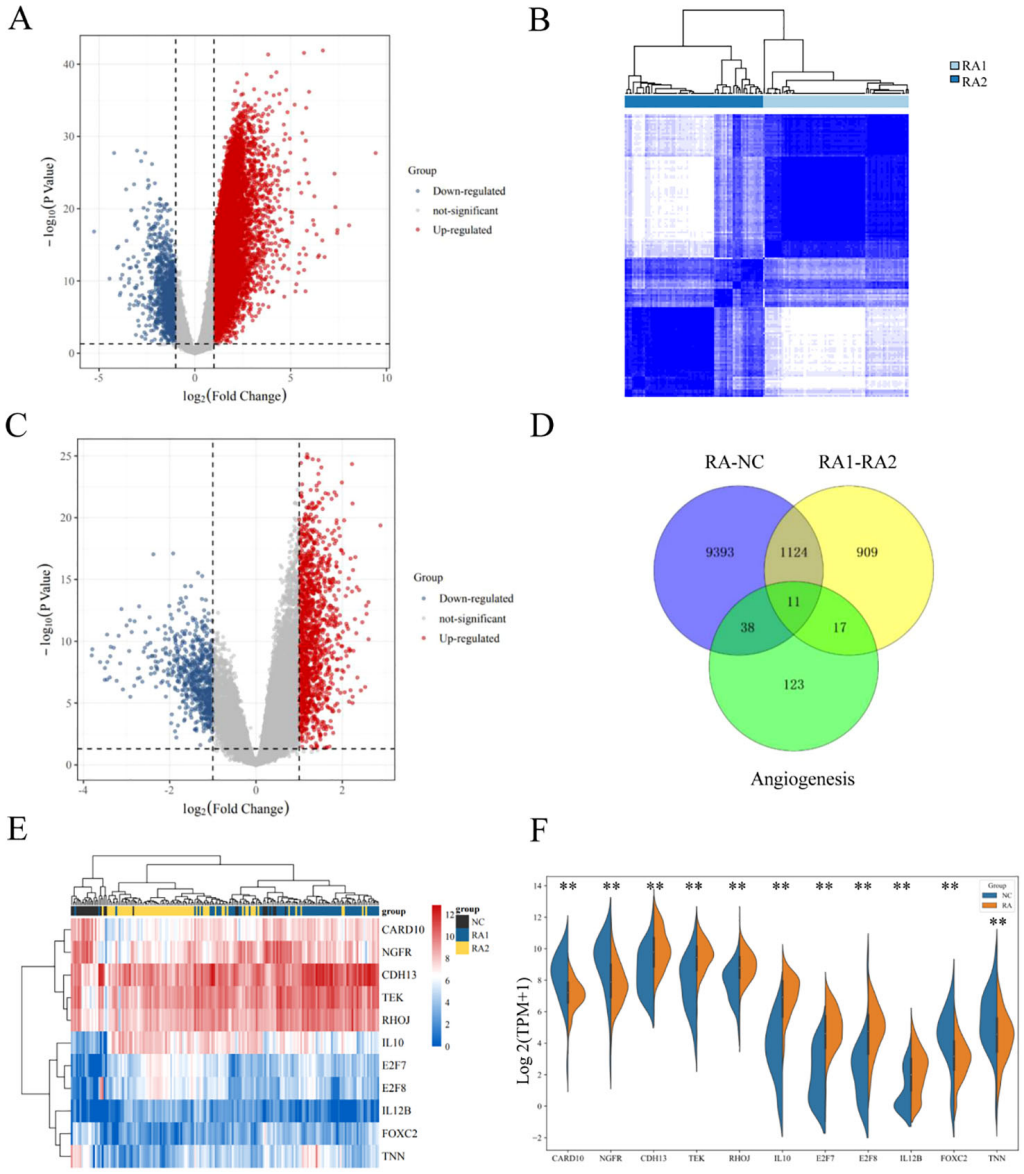
Association of RA disease progression with angiogenesis. **(A)** Volcano plot of differentially expressed RA synovial-related genes. **(B)** Subgroup clustering map of RA-related genes. **(C)** Volcano plot of differentially expressed angiogenesis-related genes. **(D)** Intersection analysis of RA-related genes, RA subgroup-related genes, and angiogenesis-related genes. **(E)** Clustering map of 11 intersecting RA angiogenesis-related genes within the group. **(F)** Expression analysis of 11 intersecting RA angiogenesis-related genes within the group. NC, Healthy Control Group; RA, Rheumatoid Arthritis Group. *P* < 0.01 compared to the Healthy Control Group. **P < 0.01.

Differential analysis of the RA synovial biopsy gene expression dataset GSE89408 (**Figure 2A**) revealed that, compared to healthy controls, 3,306 genes were upregulated (log2 FC > 1, *P* < 0.05) and 3,052 genes were downregulated (log2 FC < -1, *P* < 0.05). A total of 189 angiogenesis-related genes were identified and used to construct an angiogenesis gene set through the GSEA database. Based on the expression of these angiogenesis genes, RA samples were clustered into two distinct subtypes: RA1 and RA2 groups, identified by consensus clustering based on synovial transcriptomic profiles(**Figure 2B**). Differential expression analysis between the RA1 and RA2 groups (**Figure 2C**) identified 1,382 upregulated genes (log2 FC > 1, *P* < 0.05) and 679 downregulated genes (log2 FC < -1, *P* < 0.05). The intersection of the two differential expression analyses and the angiogenesis gene set yielded 11 common genes (CARD10, NGFR, CDH13, TEK, RHOJ, IL10, E2F7, E2F8, IL12B, FOXC2, and TNN, **Figure 2D**). The expression levels of these 11 angiogenesis-related genes across different groups are shown in **Figure 2E**. Compared to healthy controls, CARD10, NGFR, FOXC2, and TNN showed decreased expression in the synovium of RA patients (*P* < 0.05), whereas CDH13, TEK, RHOJ, IL10, E2F7, E2F8, and IL12B exhibited increased expression (*P* < 0.05), **Figure 2F**.

### 3.2 Diagnostic value analysis of angiogenesis-related genes

**Figure 3:**
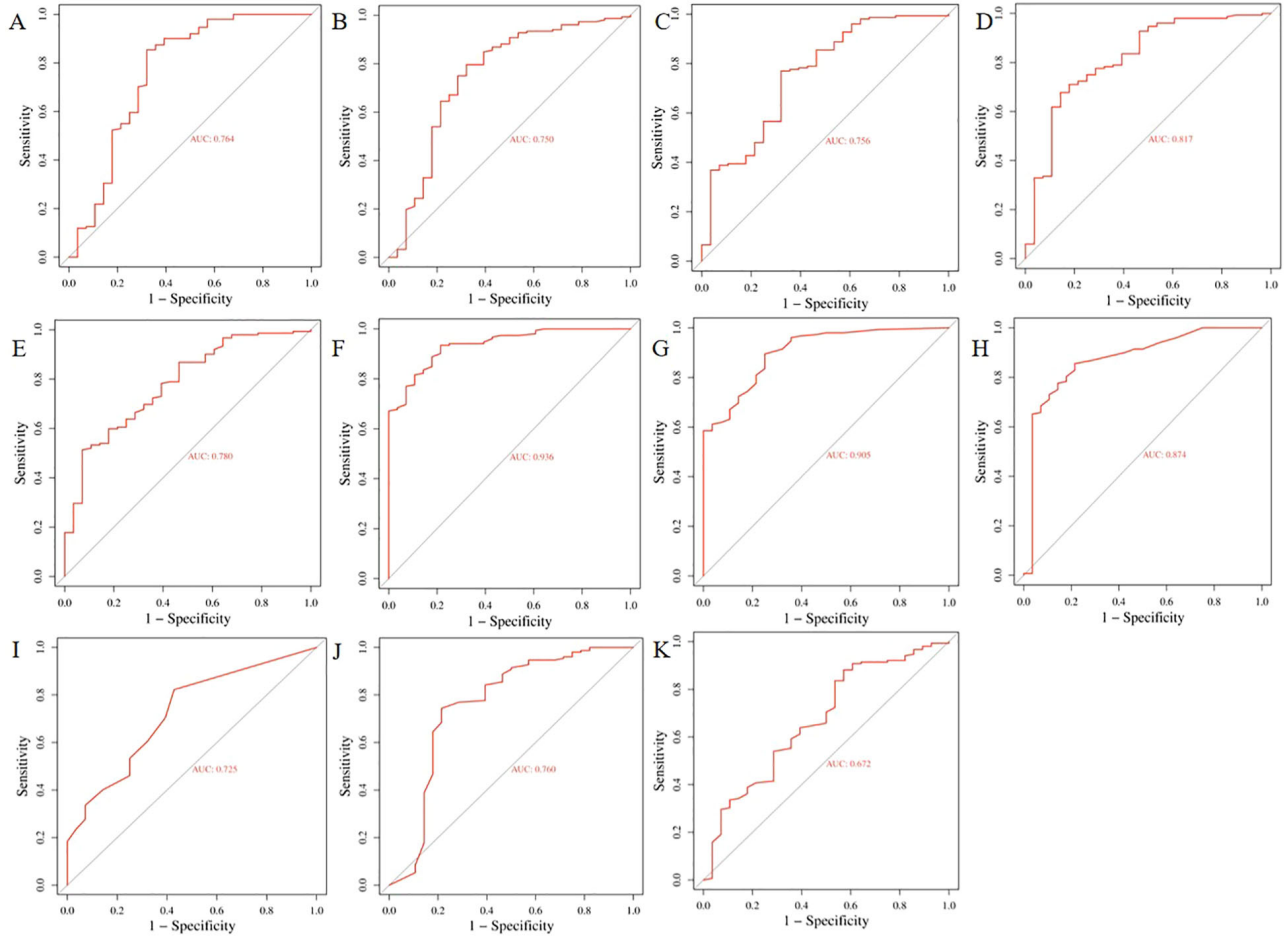
Diagnostic value analysis results of angiogenesis-related genes. **(A)** CARD10; **(B)** NGFR; **(C)** CDH13; **(D)** TEK; **(E)** RHOJ; **(F)** IL10; **(G)** E2F7; **(H)** E2F8; **(I)** IL12β; **(J)** FOXC2; **(K)** TNN.

Based on the expression data of 11 angiogenesis-related genes from the GSE89408 dataset, ROC curves were constructed using the bioinformatics online tool (https://www.bioinformatics.com.cn/) to evaluate the sensitivity and specificity of these genes for diagnosing RA. The results indicated the following AUC values for potential genes associated with RA angiogenesis: CARD10 (AUC = 0.763), NGFR (AUC = 0.750), CDH13 (AUC = 0.756), TEK (AUC = 0.817), RHOJ (AUC = 0.780), IL10 (AUC = 0.936), E2F7 (AUC = 0.905), E2F8 (AUC = 0.874), IL12B (AUC = 0.725), FOXC2 (AUC = 0.760), and TNN (AUC = 0.672). Among these, IL10, E2F7, and E2F8 exhibited high specificity in the diagnosis of RA, **Figure 3**.

### 3.3 Enrichment analysis

**Figure 4:**
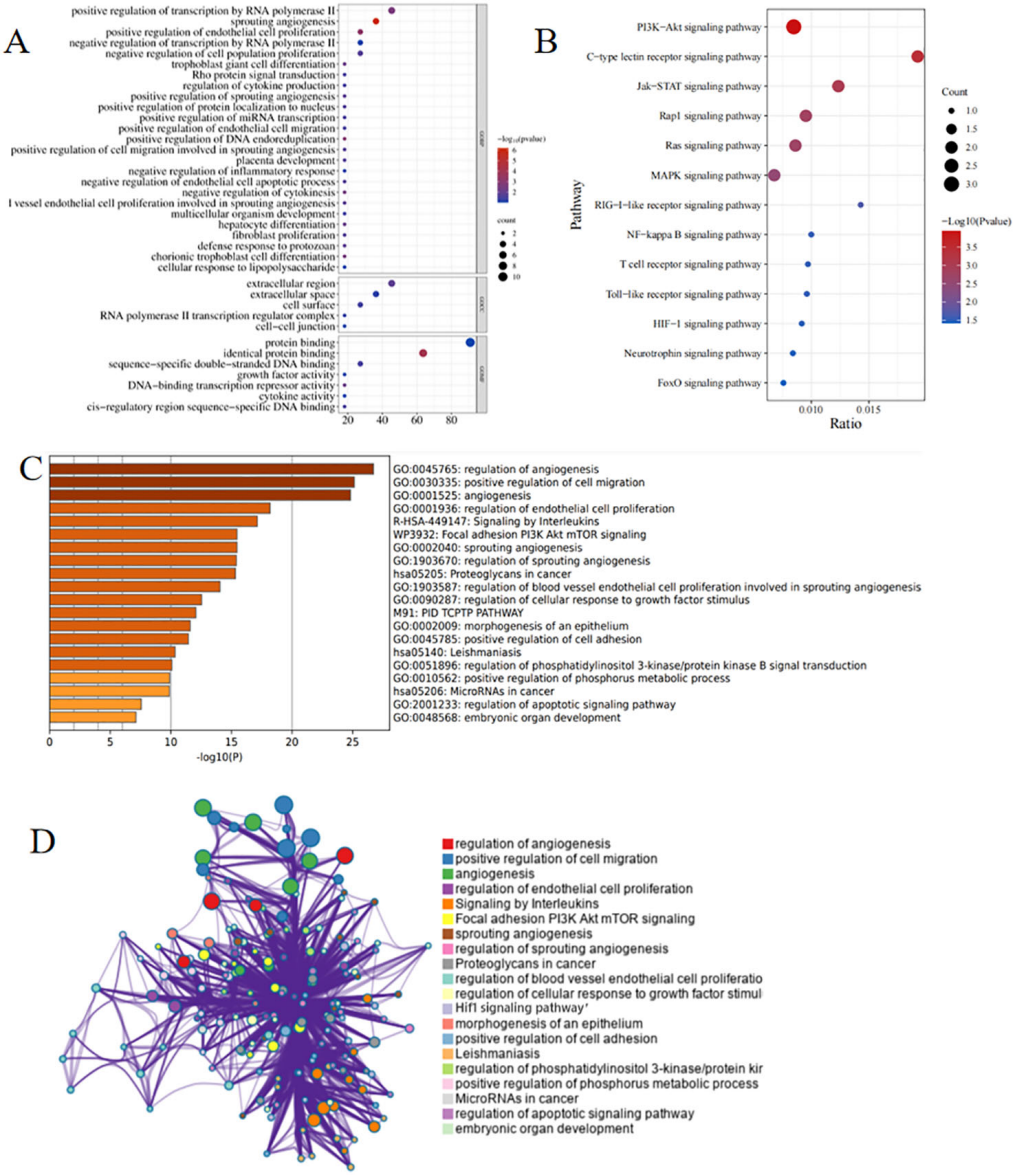
Key target enrichment analysis. **(A)** Functional Enrichment Analysis Results by DAVID **(B)** Pathway Enrichment Analysis by DAVID **(C)** Functional Enrichment Analysis Results by Metascape **(D)** Pathway Enrichment Analysis by Metascape.

To enhance the credibility of the results, we employed two distinct tools for GO and KEGG enrichment analysis (**Figure 4**). The GO functional enrichment analysis of the 11 angiogenesis-related genes using DAVID is illustrated in **Figure 4A** and Metascape in **Figure 4C**. Within the biological process (BP) category, these genes were enriched in functions such as positive regulation of endothelial cell proliferation and sprouting angiogenesis, both of which are directly related to angiogenesis and inflammation. This suggests that these genes may contribute to abnormal angiogenesis in RA by promoting endothelial cell proliferation, resulting in synovial hyperplasia and sustained inflammatory responses. Additional enrichments included positive regulation of cell migration and cell-cell signaling, consistent with previous findings indicating that cell migration in an angiogenic and inflammatory environment is crucial for the accumulation of inflammatory cells in the synovium, maintaining the local pathological state.

In terms of cellular component (CC), the genes were primarily enriched in the extracellular space, indicating that their expressed products may exert regulatory effects on angiogenesis and synovial proliferation via extracellular signaling. For molecular function (MF), significant enrichment was observed for cytokine activity and protein binding, consistent with the overexpression of cytokines and the complex protein interaction networks implicated in RA pathology. These functions may influence synovial angiogenesis and immune cell infiltration processes.

The KEGG pathway enrichment analysis revealed that these genes are significantly involved in several key signaling pathways, including the PI3K-Akt, JAK-STAT, Ras, MAPK, HIF1, and Toll-like receptor pathways. These pathways play essential roles in the regulation of angiogenesis, with the HIF1 signaling pathway being particularly notable for its role in promoting neovascularization under hypoxic conditions (**Figures 4B, D**).

### 3.4 Immune infiltration analysis

**Figure 5:**
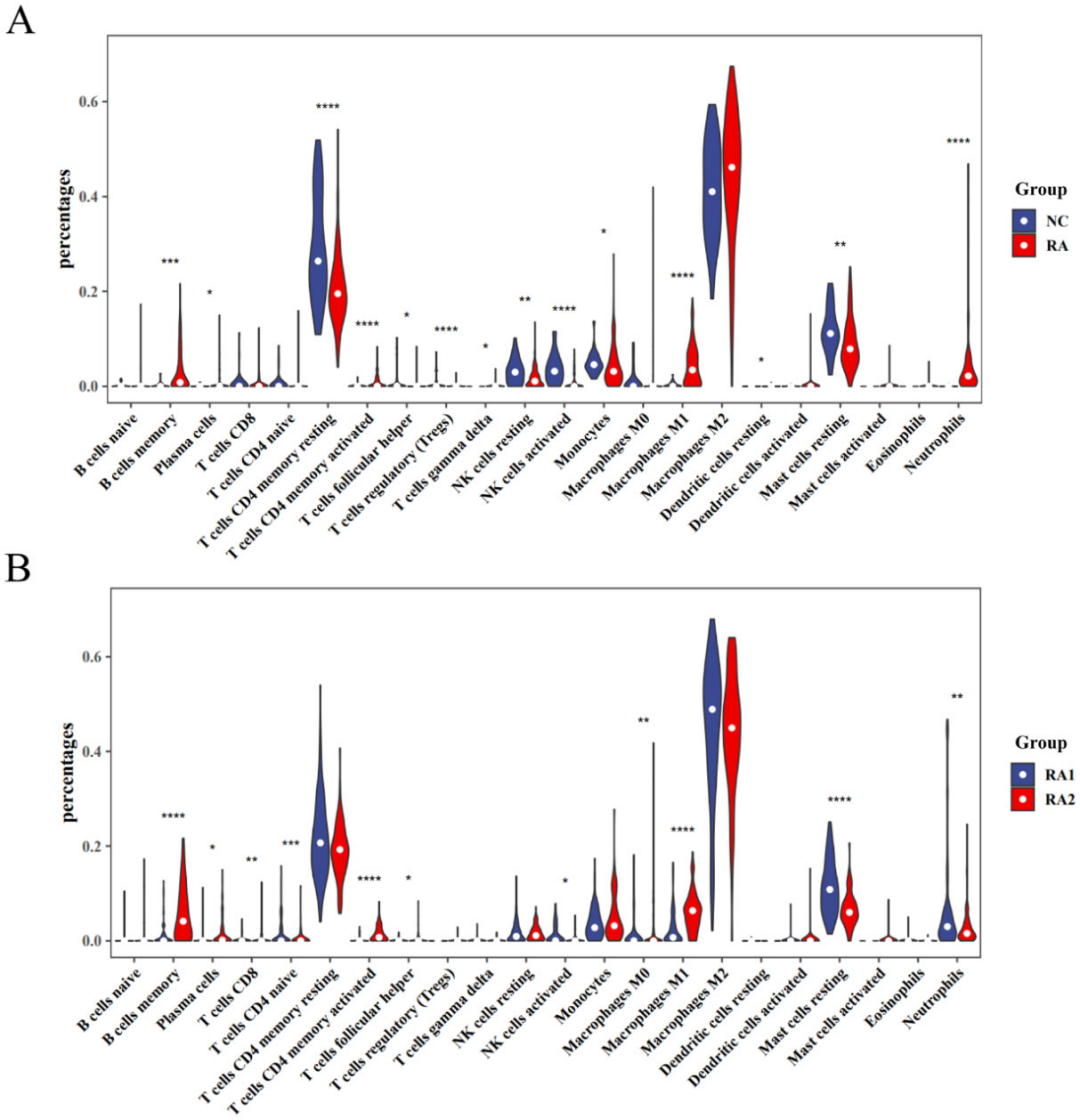
Immune infiltration analysis results. **(A)** Immune infiltration analysis of synovial samples in the RA group **(B)** Immune infiltration analysis between RA1 and RA2 groups. Note: NC: Healthy control group; RA: Rheumatoid arthritis group. Compared with the healthy control group & RA1 group: ^*^P < 0.05, ^**^P < 0.01, ^***^P < 0.001, ^****^P < 0.0001.

The immune infiltration analysis results showed that, compared to the normal control group (**Figure 5A**), synovial samples from the RA group exhibited significant differences in the abundance of memory B cells (*P* < 0.001), plasma cells (*P* < 0.05), resting memory CD4^+^ T cells (*P* < 0.0001), activated memory CD4^+^ T cells (*P* < 0.0001), follicular helper T cells (*P* < 0.05), regulatory T cells (*P* < 0.0001), γδT cells (*P* < 0.05), resting NK cells (*P* < 0.01), activated NK cells (*P* < 0.0001), monocytes (*P* < 0.05), M1 macrophages (*P* < 0.0001), resting dendritic cells (*P* < 0.05), resting mast cells (*P* < 0.01), and neutrophils (*P* < 0.0001).

Immune infiltration analysis between RA1 and RA2 groups revealed significant differences in the abundance of memory B cells (*P* < 0.0001), plasma cells (*P* < 0.05), naive CD8^+^ T cells (*P* < 0.01), naive CD4^+^ T cells (*P* < 0.001), activated memory CD4^+^ T cells (*P* < 0.0001), follicular helper T cells (*P* < 0.05), activated NK cells (*P* < 0.05), M0 macrophages (*P* < 0.01), M1 macrophages (*P* < 0.0001), mast cells (*P* < 0.0001), and neutrophils (*P* < 0.01), (**Figure 5B**).

### 3.5 Correlation analysis

**Figure 6:**
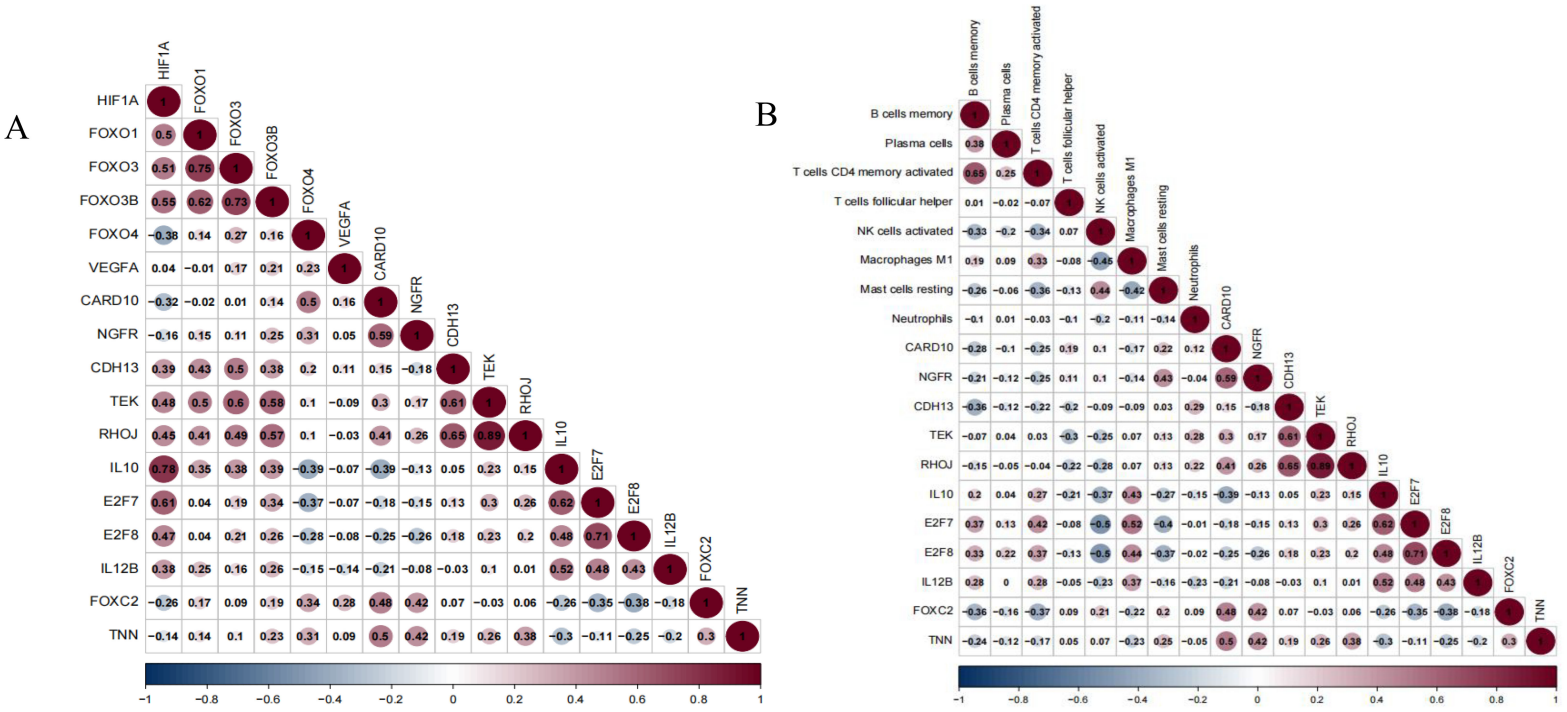
Correlation analysis results. **(A)** Correlation analysis of HIF1, FOXOs, and VEGFA with 11 angiogenesis-related genes **(B)** Correlation analysis of HIF1, FOXOs, and VEGFA with common differential immune cells.

Correlation analysis results for HIF1, FOXOs, and VEGFA with the 11 angiogenesis-related genes are shown in **Figure 6A**. HIF1 showed a positive correlation with RA-specific angiogenesis genes IL10, E2F7, and E2F8. FoxO1, FoxO3, and FoxO3B also demonstrated positive correlations with IL10, E2F7, and E2F8, although with lower correlation coefficients. In contrast, FoxO4 and VEGFA exhibited a negative correlation with the expression levels of IL10, E2F7, and E2F8. Correlation analysis of HIF1, FoxOs, and VEGFA with common differential immune cells (including memory B cells, plasma cells, activated memory CD4+ T cells, follicular helper T cells, activated NK cells, M1 macrophages, mast cells, and neutrophils) is illustrated in **Figure 6B**. The correlation coefficients between HIF1, FoxOs, VEGFA, and immune cells were generally low.

### 3.6 Single-cell data filtering and expression

**Figure 7:**
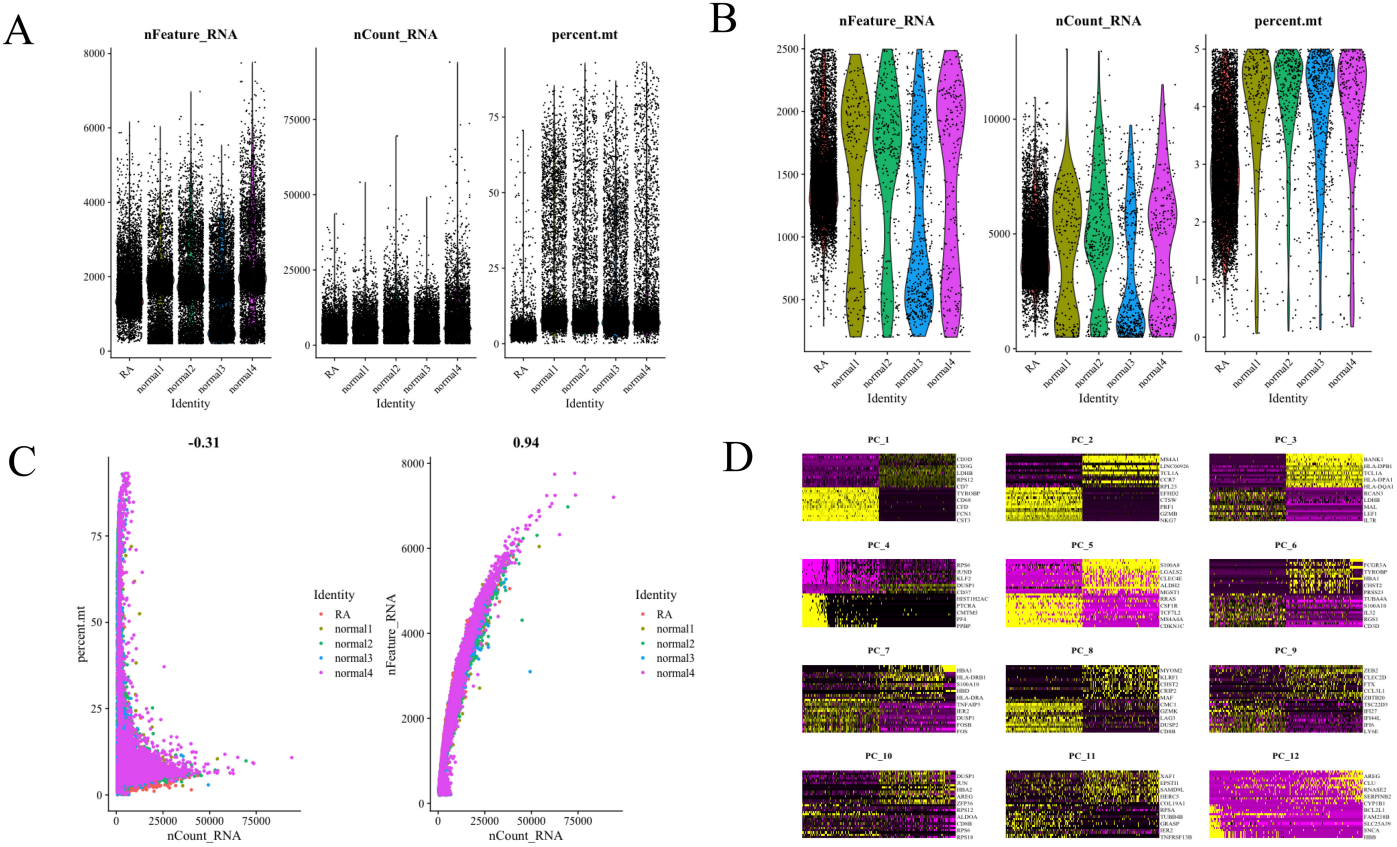
Single-cell data filtering and analysis. **(A)** Distribution of genes across groups before cell filtering. **(B)** Distribution of genes across groups after cell filtering. **(C)** Correlation among RNA expression features. **(D)** Principal Component Analysis (PCA) showing sample group separation.

The single-cell RNA sequencing dataset GSE159117 was obtained from the GEO database and subjected to quality control. Cells with a mitochondrial gene content greater than 5%, or those expressing fewer than 200 genes or more than 2,500 genes, were filtered out to eliminate low-quality cells and retain high-confidence data (**Figures 7A, B**).

Preliminary analysis revealed differences between RA and normal control samples in terms of the number of detected genes, total RNA counts, and percentage of mitochondrial gene expression, indicating substantial heterogeneity in cell characteristics across samples. After filtering, RA samples showed higher mean and range values of both nCount_RNA and nFeature_RNA compared to controls. Further correlation analysis among quality control metrics revealed a negative correlation between percent.mt and nCount_RNA (*r*=–0.31), suggesting that the proportion of mitochondrial genes decreases with increased total RNA expression. A strong positive correlation was also observed between nFeature_RNA and nCount_RNA (*r* = 0.94), indicating that the number of expressed genes increases with total RNA abundance (**Figure 7C**).

In addition, Principal Component Analysis (PCA) was performed on all genes in the dataset. The results showed a clear separation between RA and control samples, particularly along the PC1 and PC2 axes, indicating distinct gene expression patterns between the two groups (**Figure 7D**).

### 3.7 Cell-type annotation of core genes

**Figure 8:**
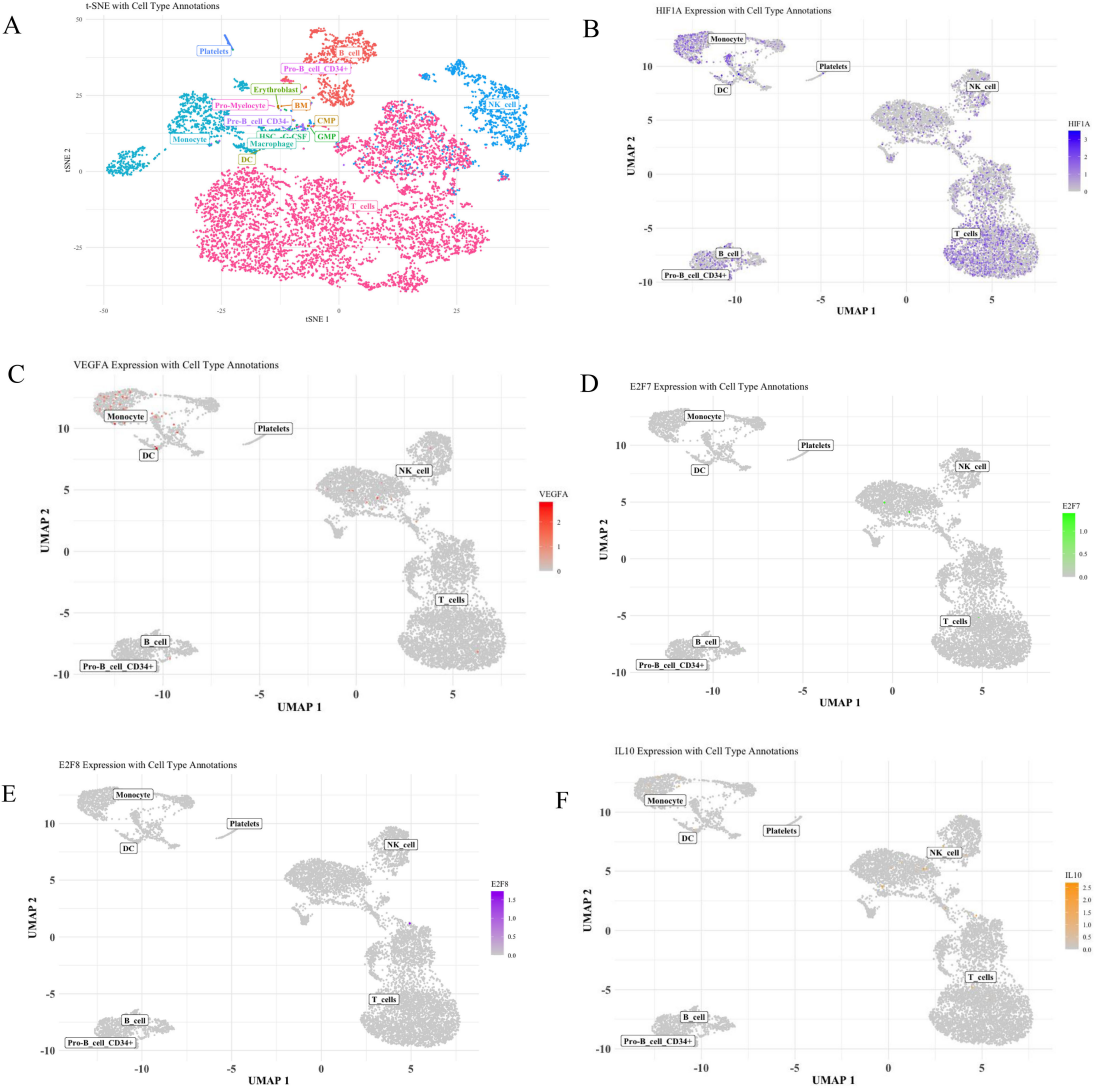
Annotation of cell types and core genes. **(A)** t-SNE plot of cell type annotation. **(B)** Annotation of HIF1 expression in cells. **(C)** Annotation of VEGFA expression in cells. **(D)** Annotation of E2F7 expression in cells. **(E)** Annotation of IL10 expression in cells. **(F)** Annotation of E2F8 expression in cells.

t-SNE dimensionality reduction and clustering analysis, along with gene-based cell type annotation, revealed heterogeneity in cell composition between RA and normal control samples, as well as differences in the distribution of specific cell types within RA (**Figure 8A**). Through single-cell transcriptomic analysis, five core genes included HIF1, VEGFA, E2F7, E2F8, and IL-10—were identified and visualized using UMAP to show their expression across different cell types (**Figures 8B–F**).

Cell-type annotation results indicated that HIF1 and VEGFA were highly expressed in monocytes and dendritic cells, suggesting their involvement in angiogenesis and hypoxic responses in the RA synovium. E2F7 and E2F8 showed low expression across most cell populations, with E2F7 displaying partial expression in monocytes, while E2F8 exhibited generally low and non-specific expression, implying reduced activity or broad, non-cell-specific function in these cells. IL-10 was predominantly expressed in monocytes, indicating its potential anti-inflammatory role in RA through modulation of immune responses and secretion of IL-10 to mitigate inflammation.

### 3.8 Validation of core gene expression by RT-qPCR

**Figure 9:**
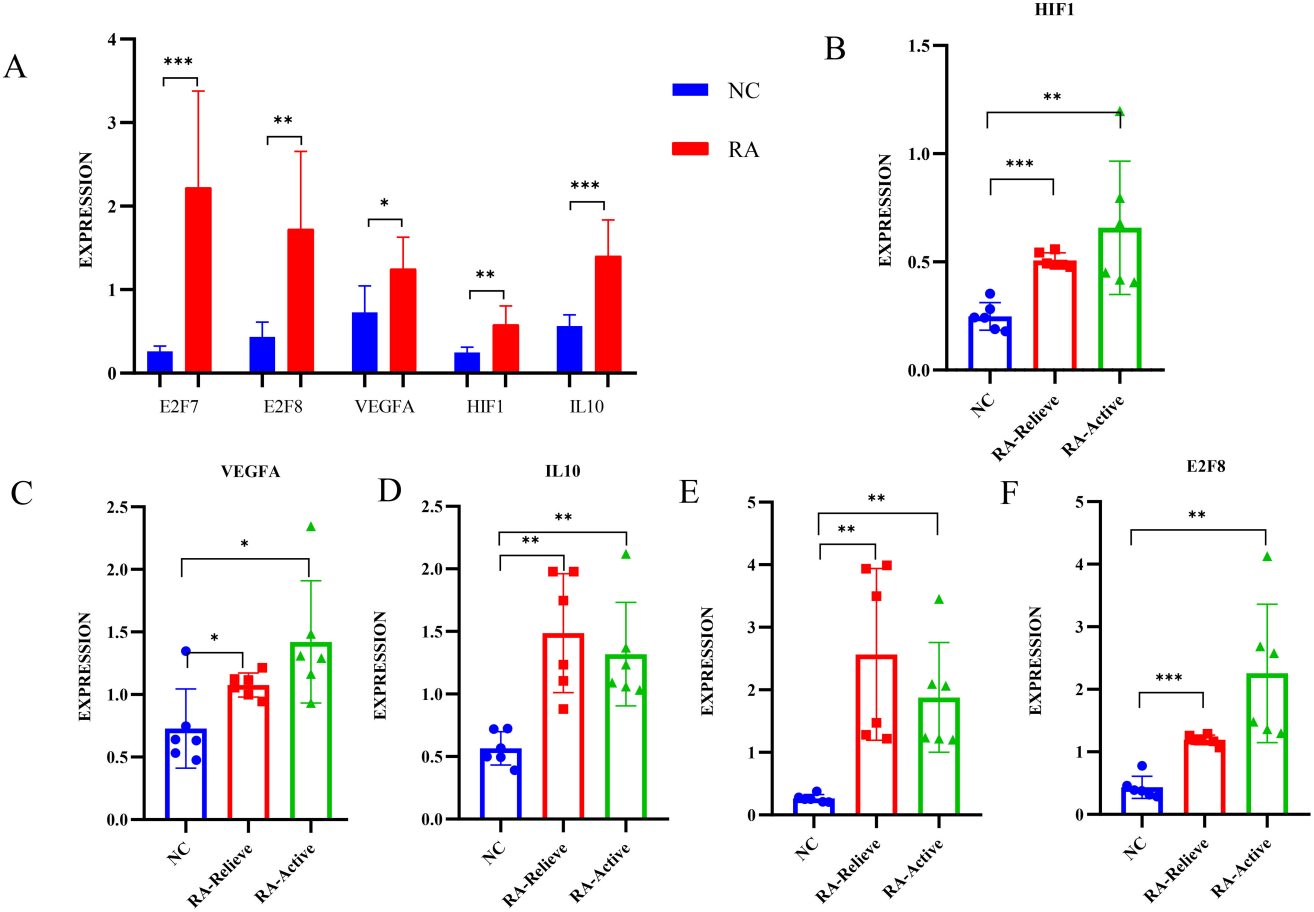
mRNA expression levels of core genes in PBMCs from RA patients and controls. **(A)** Comparison of mRNA expression levels of core genes (E2F7, E2F8, IL10, HIF1, and VEGFA) between the healthy control group and RA group. **(B–F)** Differential mRNA expression of HIF1 **(B)**, VEGFA **(C)**, E2F7 **(D)**, E2F8 **(E)**, and IL10 **(F)** among the healthy control group, RA remission group, and RA active disease group. NC, Healthy Control Group; RA, Rheumatoid Arthritis Group. ^*^P < 0.05, ^**^P < 0.01, ^***^P < 0.001 compared to the Healthy Control & RA Groups.

The baseline characteristics of the two patient groups are summarized in **Table 2**. The mRNA expression levels of the core genes E2F7, E2F8, IL-10, HIF1, and VEGFA in peripheral blood mononuclear cells (PBMCs) from healthy controls and RA patients are shown in **Figure 9**.

**Table 2:** The baseline characteristics of the two patient groups.

Label	Total (n = 18)	NC group (n = 6)	RA group (n = 12)	Statistic	P
Years	54.61 ± 12.03	51.83 ± 13.45	56.00 ± 11.63	*t* = -0.68	0.51
RF(IU/ML)	125.78 ± 10.16	NA	106.17± 13.98	*t* = -2.25	0.04
ESR(mm/h)	69.50 ± 10.02	NA	59.25 ± 14.62	*t* = -5.93	<.01
CRP(mg/L)	24.75 ± 10.18	NA	16.12 ± 28.45	*t* = -1.37	0.19
TJC	5.94 ± 3.99	NA	5.92 ± 6.54	*t* = -3.13	<.01
SJC	5.78 ± 2.15	NA	4.17 ± 5.89	*t* = -1.71	0.11
Grade	3.89 ± 2.20	NA	4.33 ± 2.99	*t* = -5.01	<.01
DAS-28	3.20 ± 2.82	NA	4.79 ± 1.99	*t* = -8.35	<.01
CCP	102.00 ± 100.97	NA	153.00 ± 85.12	*t* = -4.34	<.01
Disease duration (Month)	NA	NA	46.5 (35.5, 56.5)	NA	NA
SEX					
Male	13 (72.22)	4 (66.67)	9 (75.00)		
Female	5 (27.78)	2 (33.33)	3 (25.00)		

The expression levels of E2F7, E2F8, IL-10, HIF1, and VEGFA were significantly higher in RA patients compared to healthy controls (*P* < 0.05), which is consistent with the expression profiles observed in the RA transcriptomic dataset (**Figure 9A**).

Furthermore, based on DAS28 scores, RA patients were stratified into remission and active disease groups, and the expression of the core genes was analyzed accordingly (**Figures 9B–F**). The expression levels of HIF1, VEGFA, and E2F8 were significantly elevated in the active RA group compared to the remission group, and showed a positive correlation with disease activity.In contrast, E2F7 and IL-10 expression levels were significantly lower in the active group than in the remission group, although both remained significantly higher than in the healthy controls (*P* < 0.05).

These results further support the role of these core genes in RA pathogenesis and their potential utility as biomarkers for disease activity.

### 3.9 Clinical correlation analysis

The correlation between mRNA expression levels of the five core genes (E2F7, E2F8, VEGFA, HIF1, and IL10) and clinical parameters in RA patients was assessed.

E2F7 expression showed weak correlations (*r* < 0.5, *P*>0.05) with all evaluated clinical indices, including RF, anti-CCP, ESR, CRP, TJC, SJC, PGA, and DAS28. Moderate correlations were observed with RF (*r* = 0.49) and DAS28 (*r* = 0.43), although they were not statistically significant. Correlations with the remaining parameters were weak (*r* < 0.3, *P*>0.05). In contrast, E2F8 expression showed a strong correlation with most clinical indices (*r*>0.5, *P* < 0.05), including anti-CCP, ESR, CRP, TJC, SJC, PGA, and DAS28. Notably, E2F8 showed the strongest correlation with DAS28 (*r* = 0.80, *P* < 0.001). Only a weak correlation with RF was observed (*r* = 0.15, *P* > 0.05).

VEGFA expression was weakly correlated with RF (*r* = 0.23, *P*>0.05), moderately correlated with TJC (*r* = 0.48) and SJC (*r* = 0.49, *P* < 0.05), and strongly correlated with anti-CCP, ESR, CRP, PGA, and DAS28, with the strongest correlation observed for DAS28 (*r* = 0.67, *P* < 0.01).

HIF1 expression was weakly correlated with RF (*r* = 0.25, *P*> 0.05), moderately correlated with TJC (*r* = 0.47) and SJC (*r* = 0.46), and showed strong correlations with anti-CCP, ESR, CRP, PGA, and DAS28 (*P* < 0.05), among which DAS28 demonstrated the strongest association (*r* = 0.71, *P* < 0.01).

**Figure 10:**
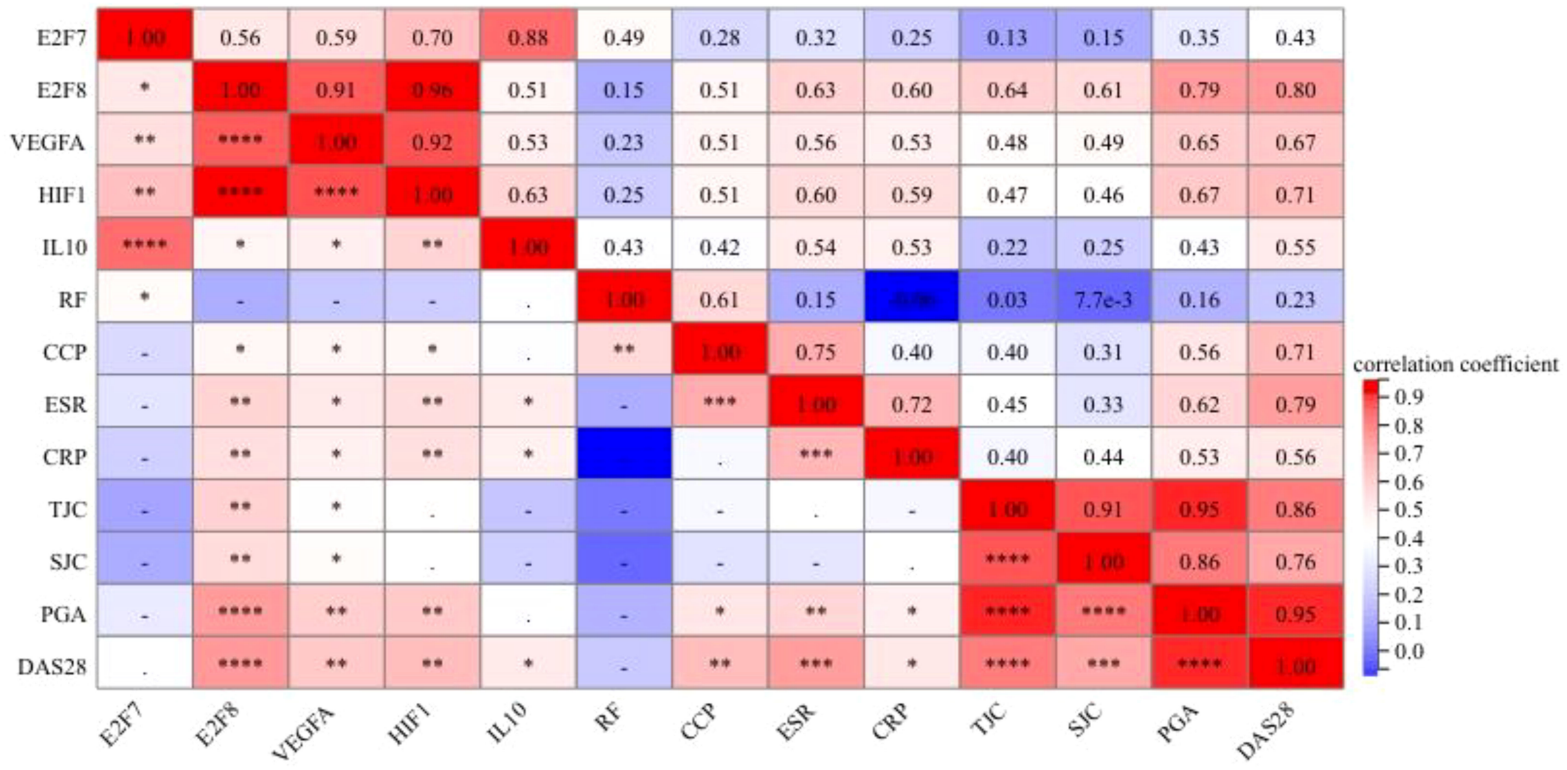
Heatmap of correlation between core gene expression and clinical parameters in RA patients. *P* < 0.05, *P* < 0.01, *P* < 0.001 vs. healthy controls.

IL10 expression exhibited moderate correlations with RF and PGA (*r* = 0.43 for both, *P*>0.05), weak correlations with TJC (*r* = 0.22) and SJC (*r* = 0.25), and strong correlations with anti-CCP, ESR, CRP, and DAS28 (*P* < 0.05), with the highest correlation again observed for DAS28 (*r* = 0.55, *P* < 0.05). The full correlation results are shown in **Figure 10**.

These findings suggest that the mRNA expression levels of the five core genes—HIF1, E2F7, E2F8, VEGFA, and IL10—are all strongly correlated with DAS28, and moderately correlated with inflammation markers ESR and CRP, while correlations with RF were consistently weak. This indicates that these core genes may reflect RA disease activity and hold potential as auxiliary biomarkers for assessing disease severity. They may provide clinicians with more precise tools to monitor disease progression and evaluate therapeutic responses in RA.

## 4 Discussion

### 4.1 Core genes drive aberrant angiogenesis in RA synovial tissue

This study identified HIF1, VEGFA, E2F7, E2F8, and IL-10 as the core genes associated with angiogenesis in RA, and the HIF1 signaling pathway was significantly expressed in RA-related angiogenesis.

Previous study found that HIF1 induces the expression of the downstream gene VEGFA, thereby promoting angiogenesis to alleviate the hypoxic condition (17, 18). VEGFA is a key downstream effector of HIF1, and its role is to promote the proliferation and migration of endothelial cells, thereby facilitating the formation of new blood vessels (19, 20). E2F7 and E2F8, members of the E2F family of transcription factors, are key regulators of the cell cycle (16). Previous studies typically classify E2F family members as transcriptional repressors and consider them inhibitors of angiogenesis. However, some studies suggested that E2F7/8 directly bind to and stimulate VEGFA expression independent of the typical E2F binding factors. Instead, E2F7/8 form a transcriptional complex with HIF1 to stimulate VEGFA activity, thereby inducing angiogenesis (5, 21). These findings reveal an unexpected link between E2F7/8 and the HIF1-VEGFA pathway, providing insights into the molecular mechanism by which E2F7/8 regulate angiogenesis.

Functional enrichment analysis revealed that HIF1 and VEGFA are highly enriched in processes such as the positive regulation of endothelial cell proliferation and sprouting angiogenesis, further confirming their core role in angiogenesis regulation in RA synovium. HIF1, MAPK, and JAK-STAT are significantly enriched in RA angiogenesis. It has been demonstrated that the HIF1 signaling pathway plays a central role in angiogenesis and the hypoxic response (22, 23). HIF1 senses the hypoxic state in RA synovium and initiates the expression of VEGFA. It activates the PI3K-Akt and MAPK signaling pathways, promoting endothelial cell proliferation and subsequently driving the formation of new blood vessels (24, 25). VEGFA regulates angiogenesis through the PI3K-Akt and JAK-STAT signaling pathways, enhancing vascularization of the synovial tissue. It also promotes abnormal synovial cell proliferation, exacerbating synovial thickening and inflammatory responses in RA (26, 27). Previous studies on the role of E2F7 and E2F8 in RA are limited, but their involvement has been significant in other inflammatory diseases (28, 29). Based on this study, we have reason to believe that E2F7 and E2F8 may participate in pathological cell proliferation in RA through cell cycle regulatory pathways, driving the excessive proliferation of synovial fibroblasts and endothelial cells, which subsequently leads to synovial hyperplasia and pathology, playing a role in the regulation of RA inflammation. The action of IL-10 is primarily mediated through the JAK-STAT signaling pathway, where it regulates the inhibition of pro-inflammatory cytokines (30, 31). However, in RA patients, despite elevated IL-10 levels, the persistent activation of other pro-inflammatory factors may weaken its anti-inflammatory effects, allowing inflammation to continue.

Overall, HIF1, VEGFA, E2F7, E2F8, and IL-10 work together driving a complex network of angiogenesis in RA synovium.

### 4.2 Core gene-immune cell regulatory in RA

We found that 5 key genes can jointly participate in the pathogenesis of RA through their interactions with immune cell populations such as monocytes and dendritic cells. This is consistent with the theories of previous studies.

The role of HIF1 in immune cells may also promote immune cell function in hypoxic environments, allowing these cells to maintain their activity under low oxygen conditions (32). Studies have shown that HIF1 can enhance the antigen-presenting ability of monocytes and dendritic cells, thereby boosting immune responses and driving the progression of RA (33). Especially in the synovial tissue with extensive infiltration of monocytes and dendritic cells, the role of VEGFA further promotes the local immune response (34, 35). Our analysis also revealed a significant increase in the expression of VEGFA in monocytes and dendritic cells in RA synovium, which is consistent with previous studies.

Previous studies have suggested that E2F7 and E2F8 may play a crucial role in the inflammatory microenvironment by intervening in T cell-related cytokines and inhibiting the expression of apoptosis-related genes (36, 37). Although the expression of E2F7 and E2F8 is not as prominent as that of HIF1 and VEGFA in single-cell data, they may still participate in pathological angiogenesis and synovial hyperplasia in RA through interactions with other cell cycle regulatory genes. Their synergistic action with HIF1 and VEGFA may further amplify this effect, especially in hypoxic environments. In addition, our study revealed that E2F7 and E2F8 are enriched in functions related to the positive regulation of cell migration and cell cycle, suggesting that they promote the proliferation and migration of synovial cells, driving the synovial hyperplasia and pathological repair process in RA. Additionally, the expression of these two genes in RA synovium is predominantly found in immune cells such as monocytes, indicating that they may influence the pathological progression of the synovium by regulating cell proliferation, differentiation, and functional states.

Meanwhile, we identified two angiogenesis-associated molecular subtypes in RA synovium through consensus clustering of transcriptomic profiles. These subtypes displayed distinct immune infiltration patterns, indicative of divergent angiogenesis-related inflammatory microenvironments. Owing to the limited clinical metadata available in public datasets, these molecular classifications remain exploratory rather than well-defined clinical phenotypes.

### 4.3 Core genes and clinical value in RA disease activity

This study found that the mRNA expression levels of HIF1, VEGFA, E2F7, E2F8, and IL-10 are closely associated with the clinical disease activity of RA, and the expression of HIF1, VEGFA, and E2F8 shows a significant upward trend with the increase in disease activity. This result indicates that the expression of angiogenesis-related genes is correlated with the severity of RA.

Previous studies have shown that elevated RA disease activity is often accompanied by exacerbated synovial inflammation, and the massive infiltration of immune cells such as monocytes and dendritic cells in synovial tissue further aggravates the local inflammatory response (38, 39). Inflammation leads to increased metabolism and oxygen consumption of synovial tissue, which in turn activates the HIF1 signaling pathway. HIF1 regulates the expression of genes such as VEGFA to promote angiogenesis, and the newly formed blood vessels provide nutritional support for immune cell infiltration and inflammatory factor release, ultimately exacerbating disease progression (40–42).

The expression of core genes is affected by the synovial inflammatory microenvironment in this study is a molecular-level manifestation of this vicious cycle. It suggests that detecting the expression levels of these genes may assist in the clinical evaluation of the synovial inflammatory status and disease progression risk in RA patients. For example, the high expression of HIF1 and VEGFA may serve as early warning indicators for elevated RA disease activity and active formation of synovial pannus, providing references for clinical treatment.

### 4.4 Limitations

This study has several limitations that warrant consideration. Clinical validation was performed using PBMCs rather than RA synovial tissue. Consequently, the findings may not fully capture the distinct molecular and cellular features characteristic of inflamed synovium. Nevertheless, RA is a systemic autoimmune disorder, and immune cells and cytokines originating from the inflamed joint can enter the peripheral circulation. Thus, PBMCs remain a valid and informative biological surrogate for assessing overall disease burden and activity. In addition, the relatively small sample size may constrain statistical power and limit the generalizability of the results. Moreover, although bioinformatic and expression analyses identified multiple candidate genes associated with angiogenesis in RA, no functional experiments were undertaken to validate their precise mechanistic roles.

## 5 Conclusion

Core angiogenic drivers in RA converge on HIF1, VEGFA, E2F7, E2F8 and IL-10, with monocytes and dendritic cells supplying the dominant immune input. Among them, the HIF1 pathway is the most prominently activated. Expression of all five genes tracks clinical disease activity; in particular, HIF1, VEGFA and E2F8 rise in lock-step with escalating severity. This up-regulation is molded by the inflammatory synovial milieu, indicating that the genes propel RA progression by fostering neovascularization within inflamed synovium and thereby couple angiogenic output to disease burden.

## Data Availability

The datasets presented in this study can be found in online repositories. The names of the repository/repositories and accession number(s) can be found in the article/supplementary material.

## Glossary

ANG: Angiotensin
AUC: Area Under Curve
BP: biological process
CARD10: Caspase Recruitment Domain Family Member 10
CC: cellular component
CCP: Cyclic Citrullinated Peptide
CDH13: Cadherin 13
CIBERSORT: Cell-type Identification By Estimating Relative Subsets Of RNA Transcripts
CRP: C-Reactive Protein
DAS28: Disease Activity Score 28
DEG: differential expression genes
DMARDs: Disease-Modifying Antirheumatic Drugs
E2F7: E2F Transcription Factor 7
E2F8: E2F Transcription Factor 8
ESR: Erythrocyte Sedimentation Rate
ETCM: Encyclopedia of Traditional Chinese Medicine
FC: fold change
FOXC2: Forkhead Box C2
GC: Glucocorticoid
GEO: Gene Expression Omnibus
GO: Gene Ontology
GSEA: Gene Set Enrichment Analysis
HIF1: Hypoxia-Inducible Factor-1
IL-1: Interleukin-1
Jak-STAT: Janus Kinase-Signal Transducer and Activator of Transcription
KEGG: Kyoto Encyclopedia of Genes and Genomes
MAPK: Mitogen-Activated Protein Kinase
MF: molecular function
NGFR: Nerve Growth Factor Receptor
PBMCs: Peripheral Blood Mononuclear Cells
PCA: Principal Component Analysis
PI3K-Akt: Phosphoinositide 3-Kinase-Akt
RA: RheumatoidArthritis
RF: Rheumatoid Factor
RHOJ: Ras Homolog Family Member J
SJC: Joint swelling
TCMSP: Traditional Chinese Medicine Systems Pharmacology Database and Analysis Platform
TEK: Tyrosine Kinase with Immunoglobulin-like and EGF-like Domains
TGF: Transforming Growth Factor
TJC: Joint tenderness
TNN: Tenascin-N
UMAP: Uniform Manifold Approximation and Projection
VEGF: Vascular Endothelial Growth Factor.
